# Microwave-Assisted Tissue Preparation for Rapid Fixation, Decalcification, Antigen Retrieval, Cryosectioning, and Immunostaining

**DOI:** 10.1155/2016/7076910

**Published:** 2016-10-20

**Authors:** Kazuo Katoh

**Affiliations:** Laboratory of Human Anatomy and Cell Biology, Faculty of Health Sciences, Tsukuba University of Technology, 4-12-7 Kasuga, Tsukuba, Ibaraki 305-8521, Japan

## Abstract

Microwave irradiation of tissue during fixation and subsequent histochemical staining procedures significantly reduces the time required for incubation in fixation and staining solutions. Minimizing the incubation time in fixative reduces disruption of tissue morphology, and reducing the incubation time in staining solution or antibody solution decreases nonspecific labeling. Reduction of incubation time in staining solution also decreases the level of background noise. Microwave-assisted tissue preparation is applicable for tissue fixation, decalcification of bone tissues, treatment of adipose tissues, antigen retrieval, and other special staining of tissues. Microwave-assisted tissue fixation and staining are useful tools for histological analyses. This review describes the protocols using microwave irradiation for several essential procedures in histochemical studies, and these techniques are applicable to other protocols for tissue fixation and immunostaining in the field of cell biology.

## 1. Introduction

Microwave irradiation during tissue processing markedly reduces the time required for fixation, decalcification, staining with chemical reagents, and incubation with antibodies.

Since the mid-1980s, microwave irradiation has been increasingly used in histological preparation. Microwave irradiation induces rapid oscillation of water molecules (2.45 GHz) and thus increases tissue temperature. Conventional microwave devices irradiate tissues both rapidly and uniformly, and microwave irradiation protocols differ according to the specific microwave devices used.

Microwave irradiation is routinely applied for special staining [1–12]. Microwave irradiation has also been applied during fixation [13] and subsequent staining procedures, such as enzyme-based staining and immunofluorescence staining.

During preparation of tissues for immunohistological studies, many artifacts that disrupt the original signals may occur, most of which are commonly associated with late fixation or low fixative volume. Late preparation of tissues causes decomposition of proteins, resulting in a lack of certain epitopes. Disruption of proteins during fixation adversely affects the epitope-antibody reaction during immunohistochemistry. Moreover, morphological changes also occur during fixation of cryosections and/or samples for electron microscopy. Conventional fixation may also result in shrinkage of tissues, such as skeletal or smooth muscle cells, or of cultured cells due to insufficient penetration of fixative (e.g., formalin solution) to completely fix tissues, and a long time is needed for fixation.

Microwave irradiation can be used to achieve more rapid fixation, solution processing, and immunostaining [13–38]. Microwave irradiation is also applied for fluorescence in situ hybridization (FISH) analysis of paraffin-embedded tissues [39–41]. Recently, the author described microwave-irradiated blood vessel fixation and immunofluorescence microscopy [42]. In this case, microwave irradiation was used to increase penetration of fixatives. The use of microwave irradiation also reduced nonspecific binding of fluorescently labeled antibodies when fixed samples were immunostained. Rapid tissue fixation and immunofluorescence staining of cultured cells using microwave irradiation have also been described [43]. Microwave irradiation was shown to significantly reduce the required incubation times with primary and secondary antibodies in immunofluorescence microscopy. We utilized a technique involving exposure of cultured cells to intermittent microwave irradiation during fixation, which resulted in good preservation of tissue immunoreactivity compared with conventional fixation, along with reduced fixation time [43].

Another issue affecting histological analysis is the effect of pretreating hard tissues, such as bone, which requires decalcification after fixation to soften the tissue and allow it to be cut using a microtome. A long time is also required to remove fat from some tissues. Conventional decalcification requires a period of about 1-2 weeks, which prevents early diagnosis in histological research [44, 45]. Tissue preparation for electron microscopy, which involves fixation and subsequent solution treatment, is also problematic. Fixation using formalin-based fixatives causes tissue shrinkage. Solution treatment, such as dehydration by passage through an alcohol series, requires a relatively long time in conventional protocols.

Conventional antigen retrieval was generally performed using an autoclave chamber at high temperature (~121°) and high pressure and always caused tissue disruption and removal from the slides. Microwave irradiation is also highly applicable for antigen retrieval on paraffin-embedded tissue sections [46–49].

Microwave tissue processing markedly reduces the processing time required for enzyme reaction, peroxidase processing, and blocking procedures. Microwave irradiation reduces the processing time to 1/3–1/10 compared to that of conventional procedures. Moreover, microwave irradiation yields low-background, high-contrast images due to the reduced nonspecific binding of staining solution or antibodies for immunofluorescence staining.

Several microwaves that allow user-selectable control of irradiation power from 150 to 400 W are available. It is also possible to precisely control the temperature using two independent systems, for example, infrared and thermocouple temperature measurement systems.

This review describes a microwave-assisted tissue preparation protocol for tissue fixation, decalcification of bone tissue, fixation of fatty (adipose) tissues, antigen retrieval of paraffin-embedded tissues, and other techniques for which microwave irradiation is applicable. In addition, application of microwave irradiation for electron microscopy of blood vessel cells in situ is also discussed.

## 2. Application for Tissue Fixation

### 2.1. Fixation of Blood Vessels In Situ [42, 50]

Due to the difficulties associated with fixation of blood vessels, because of the shrinkage of smooth muscle tissues, there have been only a few studies using blood vessels in situ. It is very difficult to obtain good fixation of blood vessels in animals, especially endothelial cells, compared to those obtained from other organs. Perfusion of paraformaldehyde causes smooth muscle contraction according to the penetration of formalin-based fixatives. During fixation, blood vessels shrink rapidly during perfusion of paraformaldehyde solution. However, we have used microwave irradiation during fixation of blood vessels and achieved good preservation of both tissue morphology and immunoreactivity.

### 2.2. Protocol

Aortae are obtained from normal adult guinea pigs 400–600 g in body weight.

1. Perfusion with 0.85% NaCl containing heparin sodium (1 U/mL) is performed via the left ventricle, and the descending thoracic aorta, abdominal aorta, and inferior vena cava are excised.
2. Vessels are cut open along the dorsal wall and pinned onto a dental wax plate, exposing the luminal surface.
3. For light microscopy, the aorta is placed in a 100-mL beaker containing 50 mL of 2% paraformaldehyde in phosphate-buffered saline (PBS).
4. For both scanning and electron microscopy, it is recommended to use 1/2 Karnovsky's fixative (2.5% glutaraldehyde and 2% paraformaldehyde in 0.1 M sodium cacodylate buffer, pH 7.2).
5. The beaker is placed on the turntable of a microwave oven and subjected to intermittent microwave irradiation at 200 W for 5 minutes (4 s on/3 s off).

After microwave irradiation, the blood vessels are rinsed with two or three changes of PBS for 10 minutes each time without microwave irradiation. They are then cut crosswise into small segments (about 5–10 mm in length) and processed for either paraffin embedding or immunofluorescence microscopy (see Application for Immunofluorescence Microscopy).

## 3. Fixation of Cultured Cells [43]

Microwave irradiation is applicable for fixation of cultured cells. Conventional fixation of cultured cells requires at least 30–60 minutes with 1% paraformaldehyde. Microwave irradiation during fixation significantly reduces the times required for both fixation and staining for immunofluorescence microscopy. All procedures, including fixation and antibody staining, are completed within 30–45 minutes without any loss of cell morphology and without nonspecific binding of dyes.

### 3.1. Protocol

1. Cells are cultured on coverslips according to standard procedures.
2. Cells are washed quickly with three changes of PBS.
3. Cells are fixed with 1% paraformaldehyde with intermittent microwave irradiation (total 5 minutes; 4 s on/3 s off at 200 W).
4. Fixed cells are quickly rinsed three times in PBS for a total of 5 minutes without microwave irradiation followed by immunofluorescence microscopy (see Application for Immunofluorescence Microscopy).

### 3.2. Notes

Two coverslips (18 × 18 mm) are placed in plastic culture dishes 50 mm in diameter. About 4 mL of fixative is added to the culture dish.

If using culture dishes 100 mm in diameter, more than 10 coverslips (18 × 18 mm) can be fixed with 10 mL of fixative.

For microwave irradiation, the authors' laboratory uses a MI-77 type microwave irradiation device (Azumaya, Tokyo, Japan) with controllable microwave irradiation power and temperature control.

## 4. Application for Decalcification [26, 27, 33, 44, 45]

Decalcification is essential after bone fixation to obtain good paraffin sections. Decalcification of bone tissues for histological research requires a very long time, that is, 2–4 days with 10% formic acid or 1-2 weeks with 10% EDTA.

With microwave irradiation, however, the processing time can be reduced to 1/5–1/10 of the original preparation time.

### 4.1. Decalcification Solution

10% formic acid in distilled water or 10% neutral EDTA.

### 4.2. Comments

*Formic Acid Decalcification*

1. For all procedures, use intermittent microwave irradiation at 400 W (5 s on/5 s off).
2. Preparation time is reduced to 1/10 of the original procedure. For formic acid, irradiation can be performed overnight.
3. The formic acid solution temperature should not exceed 45°C.

*EDTA Decalcification*

1. Use intermittent microwave irradiation at 400 W (5 s on/5 s off).
2. Preparation time is reduced to 1/10 of the original procedure. For EDTA decalcification, irradiation can be performed for 2 or 3 days.
3. The EDTA solution temperature should not exceed 45°C.
4. Decalcification solution should be changed every day.

Procedure time should be determined by each researcher. Processing time should be modified according to the size and hardness of bones. For microwave irradiation, the authors' laboratory uses a MI-77 type microwave irradiation device (Azumaya, Tokyo, Japan) with controllable microwave irradiation power and temperature control.

## 5. Application for Immunohistochemistry [7, 17, 21, 27, 33, 36, 38, 51–53]

For immunohistochemistry experiments, avidin-biotin complex interaction (the ABC method) is generally used for detection of certain types of proteins using specific primary antibodies (Vectastain ABC Kit; Vector Laboratories, Burlingame, CA). Although it is a well-established procedure for histochemical investigations, conventional protocols for ABC method require 2-3 hours. Microwave irradiation reduces both the procedure time and background noise [51].

Deparaffinization should be performed according to standard methods. After deparaffinization, microwave irradiation can be applied. A protocol for immunohistochemistry using the ABC complex is presented below.

### 5.1. Protocol

1. Deparaffinize tissues using standard procedures without microwave treatment.
2. H_2_O_2_ treatment is performed to block endogenous peroxidase for 5 minutes with microwave treatment (intermittent irradiation; 5 s on/5 s off at 200 W).
3. Antigen retrieval should be performed in this step (see Application for Antigen Retrieval of Paraffin-Embedded Samples).
4. Wash briefly with PBS.
5. Block with blocking solution for 5 minutes with microwave treatment (5 s on/5 s off at 200 W).
6. Incubate with 1st antibody for 5 minutes with microwave treatment (5 s on/5 s off at 200 W).
7. Wash samples briefly with PBS for 10 s–1 minute without microwave treatment.
8. Incubate with biotinylated anti-mouse or anti-rabbit IgG with microwave treatment. (Secondary antibody varies according to the origin of the 1st antibody.)
9. Wash samples briefly with PBS for 1 minute without microwave treatment.
10. Incubate in Vectastain ABC solution for 3–5 minutes with microwave treatment (5 s on/5 s off at 200 W).
11. Wash samples briefly with PBS twice for 1 minute each time without microwave treatment.
12. Development of ABC complex with diaminobenzidine until staining develops without microwave treatment.

All reagents should be prepared according to the manual supplied with the Vectastain ABC Kit.

## 6. Application for Immunofluorescence Microscopy [35, 37, 42, 50, 54]

For immunofluorescence microscopy, microwave irradiation reduces incubation time to about 1/5–1/10 of the original time. Our laboratory protocol for staining of guinea pig aorta and vena cava is presented below. This is an example of immunofluorescence staining of blood vessels. The following protocol should be applicable for other tissues, although the exact irradiation power and time of microwave treatment should be determined by each researcher. The detailed cultured cell protocol was reported previously [43].

For fixation using microwave irradiation (see also Application for Tissue Fixation), aortae are rinsed several times with PBS and cut into small pieces, and en face preparations are made.

### 6.1. Protocol

1. Permeabilize the tissue with 0.5% Triton X-100 in PBS for 5 minutes without microwave irradiation.
2. The specimens are then incubated with 10% normal goat serum for 5 minutes with intermittent microwave irradiation (4 s on/3 s off at 200 W).
3. The specimens are rinsed several times with PBS without microwave irradiation.
4. Incubation should then be performed with one of the primary antibodies (in this case, antipaxillin as a marker of adhesion plaques located at sites of cell-substrate adhesion) for 5 minutes with intermittent microwave irradiation (4 s on/3 s off at 200 W).
5. Rinse specimens with PBS several times without microwave treatment.
6. Incubate with FITC-labeled secondary antibody for 5 minutes with microwave irradiation (4 s on/3 s off at 200 W).
7. [Optional] Further staining with dyes can be performed, such as rhodamine-labeled phalloidin for F-actin or propidium iodide for nuclear staining with microwave irradiation (4 s on/3 s off at 200 W).
8. Mount samples on slide glasses, followed by immunofluorescence or confocal laser scanning microscopy.

The temperature of samples should not exceed 40°C for immunofluorescence microscopy.

**Figure 1:**
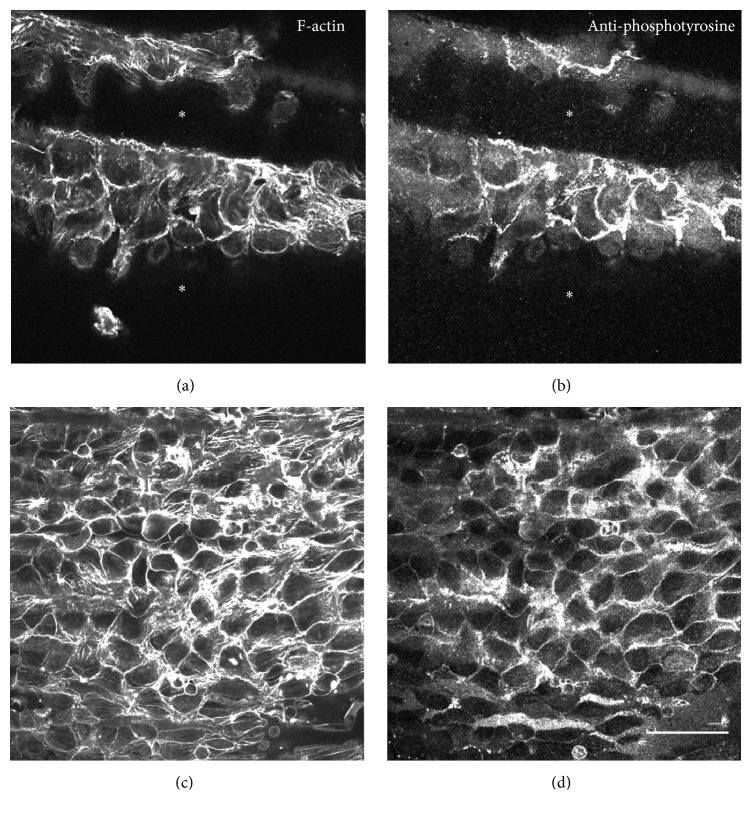
Comparison between conventional fixation (a and b) and microwave irradiation (c and d) of guinea pig aortic endothelial cells in whole-mount preparations. Conventionally fixed specimens showed shrinkage in the smooth muscle cell layer. Asterisks in (a) and (b) indicate gutters caused by shrinkage of the smooth muscle cell layer. Fixation with microwave irradiation showed well-preserved and flattened morphology of the endothelium (c and d). Samples were stained with both anti-phosphotyrosine antibody to reveal tyrosine-phosphorylated proteins (b and d) and rhodamine-labeled phalloidin for F-actin staining (a and c). Samples were observed by confocal laser scanning microscopy, and focus was adjusted at the endothelial cell layer. Bar = 10 *μ*m. See also [42].

An example of typical double staining with anti-phosphotyrosine (PY-20) antibody (BD Biosciences, Franklin Lakes, NJ) as a tyrosine-phosphorylated protein marker and rhodamine-labeled phalloidin for actin filament staining is shown in Figure 1. The above protocol is applicable for staining of cryosections (see also [42]). See also immunofluorescence microscopy of paraffin-embedded samples by other authors [35, 37].

## 7. Application for Antigen Retrieval of Paraffin-Embedded Samples [23, 37, 46–49]

In general, paraffin-embedded samples that have been fixed with paraformaldehyde are not suitable for immunohistochemical staining or in situ hybridization due to the masking of antigenic sites by protein-protein cross-linking with paraformaldehyde. In some formalin-fixed samples, antigens of certain proteins are masked according to protein cross-linking by formaldehyde. Therefore, formalin-fixed samples require antigen retrieval using special treatment. Heat-induced antigen retrieval is often used to retrieve the epitopes of proteins by autoclaving or using a commercially available pressure cooker. Heat-induced antigen retrieval sometimes causes damage to fixed samples.

Conventional procedures involved using an autoclave (121°C with pressure). In some cases, a household kitchen microwave oven (nearly 100°C) was used. In many cases, tissues on slide glasses became detached, and high levels of background noise were observed because of tissue damage by high temperature.

Microwave irradiation using a purpose-built apparatus can yield stable experimental results for research [46, 49].

### 7.1. Comments

1. Beaker with antigen retrieval solution 400 mL (e.g., Target Retrieval solution; DAKO, Produktionsvej, Denmark).
2. Maximum solution temperature: 70°C–99°C.
3. Continuous irradiation for 20–30 minutes (intermittent irradiation: 5 s on/5 s off at 400 W).
4. Precise reaction time determined by each researcher.

## 8. Application for Processing Fatty Tissues

Thymus gland, breast, and lymph node specimens are typical fatty (adipose) tissues. Fixation of fatty tissues is difficult because of the low penetration of paraformaldehyde solutions. Poor tissue fixation causes tissue destruction and “bubbles” in paraffin-embedded sections. Due to the poor penetration of paraformaldehyde solution, fatty tissues require treatment with a mixture of xylene and methanol after fixation. In general, treatment of fatty tissues requires about 10–30 hours. With microwave irradiation, however, the fixation time can be significantly reduced to about 1/20–1/30 with good fixation.

### 8.1. Procedure

Take thymus gland as an example.

Refer to Application for Tissue Fixation for tissue fixation protocol.

1. Incubate fixed fatty tissues in beaker with treatment solution: xylene : methanol = 1 : 1 (500 mL).
2. Intermittent microwave irradiation for 30–60 minutes at 400 W (5 s on/5 s off): replace treatment solution if there is sedimentation of fat.
3. During microwave irradiation, the temperature should not exceed 50°C.

Irradiation times should change according to the volume of the fatty tissues. Total irradiation time should be determined by each researcher.

## 9. Other Applications of Microwave Irradiation

### 9.1. Application for Electron Microscopy

**Figure 2:**
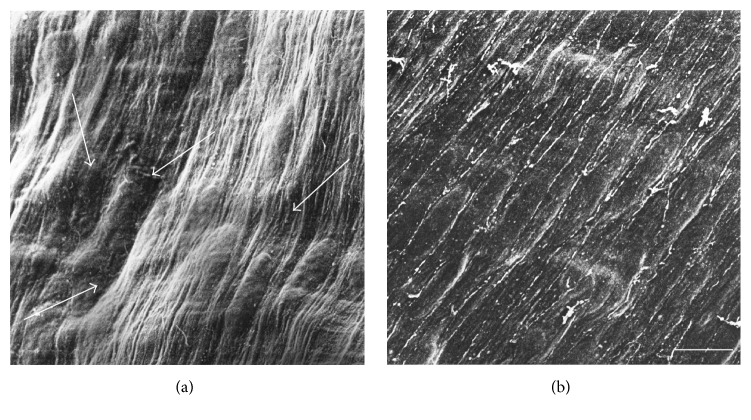
Scanning electron micrographs of venous endothelial cells without (a) or with (b) microwave irradiation. Guinea pig venous blood vessels were fixed conventionally with 1/2 Karnovsky's solution (a). Shrinkage of the smooth muscle cell layer occurred (a: arrows). After fixation with microwave irradiation, the flattened endothelial cell layer located in the inner surface of blood vessels was well-preserved (b). Arrows in (a) indicate the wavy artifacts caused by shrinkage of the smooth muscle cell layer. Compare (a) without microwave irradiation and (b) with microwave irradiation; the microwave irradiation showed good preservation of the endothelial cell layer morphology (b). Bar = 10 *μ*m. See also [55].

Tissues to be analyzed by electron microscopy are usually embedded in resin for ultramicrotomy. Although the embedding procedures vary with respect to chemical composition, they generally require about 1 week for processing. During sample preparation for electron microscopy, use of microwave irradiation reduces the procedure time to only 2 days without any loss of fine structure in tissue samples. Our microwave irradiation technique for fixation seems to be applicable to both transmission and scanning electron microscopy (see Figure 2 for SEM image).

Refer to Application for Tissue Fixation for tissue fixation protocol.

### 9.2. Protocols

(1) Postfixation with 1% osmium tetroxide in distilled water with microwave treatment for 15 minutes^*∗*^ (all procedures in this protocol use intermittent irradiation, 5 s on/5 s off at 200 W).
 (1% uranyl acetate incubation for 10 minutes with intermittent microwave irradiation if required.)
(2) Alcohol 50% with microwave irradiation for 5 minutes.
(3) Alcohol 75% with microwave irradiation for 5 minutes.
(4) Alcohol 90% with microwave irradiation for 5 minutes.
(5) Alcohol 100% with microwave irradiation for 5 minutes × 2 times.
(6) Propylene oxide 100% with microwave irradiation for 5 minutes × 2 times.

*For Transmission Electron Microscopy [56, 57]*

(7) Propylene oxide : epoxy resin = 1 : 1 with microwave irradiation for 30–60 minutes.
(8) Ероху resin 100% and microwave irradiation for 30–60 minutes.
(9) Embedding in epoxy resin according to standard procedures (60°C) for 15–20 hours, followed by thin sectioning.

*Notes*. For transmission electron microscopy, microwave irradiation also can reduce staining time with uranyl acetate and lead citrate [24, 58].

*For Scanning Electron Microscopy [55]*

(7) Transfer to 100%  *t*-butyl alcohol with microwave irradiation for 5 minutes × 2 times.
(8) Samples are freeze-dried under high vacuum.
(9) Dehydrated samples are coated using an ion-sputtering device (gold-platinum) and observed by scanning electron microscopy.

Samples with/without microwave treatment are shown in Figure 2.

### 9.3. Notes

Sample size should not exceed 2 mm (increase time for large-sized specimens >2 mm in length). Do not exceed the maximum temperature of 50°C. The exact time should be determined by each researcher. Do not exceed the maximum temperature of 50°C during the dehydration procedure using an alcohol series (50%–100%).

For postfixation in osmium tetroxide, sample temperature should not exceed 37°C when using microwave irradiation. To prevent exposure to osmium tetroxide gas, the procedure should be performed in a laboratory fume hood. Alternatively, postfixation can be performed conventionally for 1 hour at room temperature without microwave irradiation in a tightly closed screw-topped vial (1 mL of 1% osmium tetroxide solution in 10-mL vial).

## 10. Application for Cryosectioning [42]

Frozen sections are used for pathological diagnosis, enzyme detection, and immunofluorescent microscopy using antibodies. During freezing, crystallized water rapidly damages the tissues. Microwave irradiation reduces the formation of water crystals in tissues and can maintain the fine structure. Microwave irradiation mixes well with generally used Tissue-Tek OCT compound (Sakura Finetek, Tokyo, Japan) and can be sliced very easily. Compared with nonirradiated specimens, microwave-irradiated samples have fewer bubbles around the tissues and structures are well-preserved.

Refer to Application for Tissue Fixation for tissue fixation protocol.

### 10.1. Protocols

1. For cryoprotection, fixed tissues are immersed in 30% sucrose solution with microwave irradiation until the sample sinks to the bottom of the beaker (all procedures in this protocol use intermittent irradiation, 5 s on/5 s off at 200 W). Change sucrose solution 2-3 times to prevent reduction of sucrose concentration.
2. Dissect samples into small blocks (5–10 mm) using a sharp knife.
3. Immerse trimmed tissues in 10 mL of Tissue-Tek/sucrose solution (1 : 1) in a small beaker or plastic bottle with microwave irradiation for 10–15 minutes (5 s on/5 s off at 200 W).
4. Place the above tissues on parafilm (Bemis Flexible Packaging, Chicago, IL) and drop Tissue-Tek over the sample.
5. [Optional] If bubbles are observed around the tissue, the sample could be treated with microwave irradiation for 5 minutes (5 s on/5 s off at 200 W), which can remove some bubbles.
6. Place Tissue-Tek on precooled cryostat head, mount above sample, and drop Tissue-Tek over the samples as soon as possible.
7. Quickly freeze Tissue-Tek and samples with HFC134a aerosol cold spray (CRYON: Oken-Syoji, Tokyo, Japan).
8. Cut cryosections according to standard procedures.

*Comments*. When samples are mounted on the precooled cryostat head, freeze as quickly as possible to prevent formation of ice crystals in the samples.

## 11. Application for Special Staining

For special staining, such as that using periodic acid-methenamine-silver stain (PAM), Azan staining, Grimelius' method, Fontana-Masson stain, methenamine silver-nitrate Gomori-Grocott's variation, and Congo red stain, microwave irradiation allows good results without background staining within a very short time. Conventionally, the above staining procedures take 1-2 hours, while microwave irradiation allows completion of staining within 5–10 minutes [1, 3, 5, 7, 8, 59].

## 12. Conclusions

Microwave irradiation can be applied during paraformaldehyde fixation and several types of staining procedure. It also significantly reduces the time required for staining procedures, such as immunohistochemistry, immunofluorescence microscopy, and special staining of tissues. Moreover, it reduces the time required for decalcification of bone. Conventional microwave ovens are unsuitable for laboratory use because the irradiation power is too high or they do not allow precise control of the power and sample temperature. Use of a conventional microwave oven requires calibration [60]. Modern microwave devices built specifically for laboratory use allow precise control over the power of microwave irradiation and sample temperature. Microwave irradiation should be highly applicable for many histological and cell biological techniques without any loss of morphology or immunoreactivity of tissues.
